# The Causal Influence of Life Meaning on Weight and Shape Concerns in Women at Risk for Developing an Eating Disorder

**DOI:** 10.3389/fpsyg.2021.593393

**Published:** 2021-02-11

**Authors:** Sanne F. W. van Doornik, Klaske A. Glashouwer, Brian D. Ostafin, Peter J. de Jong

**Affiliations:** ^1^Department of Clinical Psychology and Experimental Psychopathology, University of Groningen, Groningen, Netherlands; ^2^Department of Eating Disorders, Accare Child and Adolescent Psychiatry, Groningen, Netherlands

**Keywords:** eating disorder, body image, meaning in life, goals, color-naming interference task, overvaluation of shape and weight

## Abstract

**Background:** Although previous studies have shown an inverse relation between life meaning and eating disorder symptoms, the correlational nature of this evidence precludes causal inferences. Therefore, this study used an experimental approach to test the causal impact of life meaning on individuals' weight and shape concerns.

**Methods:** Female students at risk for developing an eating disorder (*N* = 128) were randomly assigned to the control or the meaning condition, which involved thinking about and committing to pursue intrinsically valued life goals. A color-naming interference task was used to assess the motivational salience of body-related stimuli, and self-report measures were used to assess participants' overvaluation of weight and shape.

**Results:** The meaning manipulation was effective in activating intrinsically valued life goals. However, it did not result in lower self-reported overvaluation of weight and shape or lower color-naming interference effects of body-related stimuli, compared to the control condition. *Post-hoc* analyses suggested that baseline meaning in life was related to the impact of the manipulation.

**Conclusions:** This experimental study did not provide evidence for a causal influence of life meaning on the overvaluation of weight and shape in a high-risk group. The current findings suggest that we first need to examine the relationship between life meaning and eating disorder symptoms in more detail, before implementing brief meaning manipulations in clinical practice.

## Introduction

Eating disorders (EDs) such as anorexia nervosa and bulimia nervosa are severe mental disorders, marked by emotional distress, psychosocial impairment, and physical morbidity (Stice et al., 2013; Dakanalis et al., 2017). One out of every 10 women is at one point in her life affected by any of the EDs mentioned in the DSM-5 (Stice et al., 2013), leading to enormous health care and burden of disease costs (Schmidt et al., 2016). Despite the great impact of EDs, current treatment options are limited in their effectiveness and recurrence rates are high (e.g., Stice et al., 2013; Brockmeyer et al., 2018). Therefore, there is a great need to develop new, evidence-based treatment options that focus on the maintaining factors of EDs. One of the factors that has recently been proposed to be relevant in the development and maintenance of EDs is (reduced) meaning in life (Marco et al., 2017). In the current study, we investigated whether temporarily enhancing life meaning in a high-risk group can reduce overvaluation of weight and shape, a core symptom of EDs (Fairburn et al., 2003).

According to the leading cognitive behavioral theories of EDs (Fairburn et al., 2003; Williamson et al., 2004), overvaluation of weight and shape is considered a key transdiagnostic factor underlying the development, maintenance, and relapse of EDs. It is used as a diagnostic criterion in the DSM-5 for anorexia and bulimia nervosa (American Psychiatric Association, 2015) and has been found to differentiate eating disordered from non-disordered individuals (Goldfein et al., 2000). That is, in contrast to the more extensive meaning frameworks of non-disordered populations, the goals of individuals with EDs are narrowly focused on weight and shape (Fairburn et al., 2003). Overvaluation of weight and shape expresses itself in self-schemas that direct the person's attention to body-related stimuli and promote biased interpretations of self-relevant events (Williamson et al., 2004). Research using self-report measures showed that individuals with EDs indeed report higher levels of overvaluation of weight and shape (e.g., Geller et al., 1997; Lethbridge et al., 2011). These self-report findings are complemented by research using implicit performance-based measures, which has shown that body-related words elicited heightened color-naming interference effects in individuals with EDs (Dobson and Dozois, 2004; Johansson et al., 2005).

Overvaluing the importance of weight and shape may create a self-reinforcing loop, such that a goal to lose weight may enhance the motivational salience of goal-related cues (e.g., “fat” body parts) with the consequence that these cues grab attention, thereby leading to elaborative processing of the cues, which reactivates the weight loss goal, and so on (Fairburn et al., 2003; Williamson et al., 2004). As a result, the individual's meaning framework increasingly narrows to body-related concerns, thereby reducing the personal relevance of other values and goals. Previous studies on addictive behaviors have shown that heavy drinkers demonstrated lower incentive response to non-alcohol goal cues (Ihssen et al., 2010) and that heightened accessibility and commitment to non-alcohol life goals reduced the motivational pull of alcohol-related cues (Palfai and Weafer, 2006; Ostafin and Feyel, 2019). Activating life goals that are unrelated to weight and shape might similarly reduce the motivational salience of weight and shape concerns, thereby breaking the ED-reinforcing vicious cycle.

Research on the role of life meaning in EDs is relatively scarce (Marco et al., 2017). An overall sense of life meaning has been proposed to consist of the extent to which life is experienced as making sense (comprehension), as being directed by valued goals (purpose), and as mattering in the world (significance) (George and Park, 2016; Martela and Steger, 2016). This recently developed tripartite view highlights three fundamental dimensions of life meaning, capturing much of the variance in previous definitions of meaning (George and Park, 2017). An overall sense of life meaning is commonly assessed with unidimensional measures as the Meaning in Life Questionnaire (MLQ; Steger et al., 2006) and the Purpose in Life Test (PIL; Crumbaugh and Maholick, 1969), while the Multidimensional Existential Meaning Scale (MEMS; George and Park, 2017) can be used to assess life meaning in a multidimensional way. Although several authors have suggested that ED symptoms provide individuals with a sense of comprehension, purpose, and significance (Serpell et al., 1999; Fox and Leung, 2009; Ison and Kent, 2010), such meaning-making strategies may have dysfunctional outcomes. For example, the positive feedback loop mentioned above may narrow the individual's behavioral repertoire to goal-directed activities (i.e., striving to achieve weight and shape related goals), thereby reducing access to the diversity of common life meaning sources such as personal development, relationships, and generativity (Debats, 1999; Emmons, 2003; Schnell, 2011). A potential consequence is that in the long run, EDs may lead to a subjective sense that overall, life lacks meaning (Marco et al., 2017). In line with this idea, research has found that individuals with EDs report lower life meaning (Marco et al., 2017) and lower existential well-being (Fox and Leung, 2009). Additionally, research in individuals with EDs has shown an inverse relation between life meaning and ED symptoms, body dissatisfaction, and overweight preoccupation (Marco et al., 2017, 2019).

Although the literature consistently shows that EDs are inversely related to life meaning (Fox and Leung, 2009; Marco et al., 2017, 2019), the correlational nature of the research precludes inferences about the causal role of life meaning on EDs. Thus, a critical next step is to examine the relationship between life meaning and ED symptoms with an experimental design. Initial research has shown that a brief life meaning manipulation reduced the motivational salience of alcohol-related cues, reflected as reduced color-naming interference of alcohol-related stimuli (Ostafin and Feyel, 2019). Following a similar approach, the current study investigated whether activating life goals can reduce current overvaluation of weight and shape in a high-risk group. Specifically, we tested whether a brief meaning manipulation would lower (i) self-reported weight and shape concerns, and (ii) motivational salience of body-related stimuli, as indexed by color-naming interference of thin-related and fat-related words. As a subsidiary aim, we explored whether the meaning manipulation would also result in (iii) lower self-reported salience of thin-related and fat-related goals, and (iv) lower intention to diet.

## Materials and Methods

### Participants

An online screening survey with the Weight Concern Scale (Killen et al., 1994) was completed by 1,049 female students aged 16–30 from the University of Groningen. Participants scoring ≥ 47, indicative of high weight and shape concerns and an increased risk for developing EDs (Jacobi et al., 2004), were invited to participate in the present study [in line with Jacobi et al. (2004) each of the five items is adjusted to equal a maximum of 20: total range 0–100]. Color blind participants and participants with a history of EDs or currently in treatment for an ED were excluded. Of the 248 eligible participants, 129 participated in the current study. The data from one participant were omitted from all analyses because the screening data did not match the person showing up for the experiment. The remaining 128 participants completed the current study either in Dutch (*n* = 17; experimental condition *n* = 9, control condition *n* = 8) or English (*n* = 111; experimental condition *n* = 56, control condition *n* = 55). In return for their participation, participants received course credits (*n* = 73; experimental condition *n* = 41, control condition *n* = 32) or monetary compensation (*n* = 56; experimental condition *n* = 24, control condition *n* = 31). Participants had a mean age of 21.07 years (*SD* = 2.90) and a mean Body Mass Index (BMI; calculated based on self-reported weight and height, as kg/m^2^) of 23.55 (*SD* = 5.63). Although the mean BMI for the total sample was within the range indicative for a healthy body weight, according to international guidelines five participants classified as being underweight (BMI < 18.5, experimental condition *n* = 3, control condition *n* = 2), 17 participants were overweight (BMI between 25.0 and 29.9, experimental condition *n* = 10, control condition *n* = 7), five participants had obesity class I (BMI between 30.0 and 34.9, experimental condition *n* = 3, control condition *n* = 2), one participant had obesity class II (BMI between 35.0 and 39.9, experimental condition *n* = 1), and two participants had obesity class III (BMI over 40.0, experimental condition *n* = 2).

### Materials

#### Primary Outcome Measures

##### Overvaluation of Weight and Shape

Four items were presented to assess current overvaluation of weight and shape. The first two items (labeled as OWS-1) were derived from the Weight Concern subscale and Shape Concern subscale of the Eating Disorder Examination Questionnaire (EDE-Q; Fairburn and Beglin, 2008): “Right now, my weight influences how I think about (judge) myself as a person” and “Right now, my shape influences how I think about (judge) myself as a person.” We adapted the time frame of these items by adding the words “right now” to measure momentary overvaluation of weight and shape. Participants rated the items from 0 (strongly disagree) to 6 (strongly agree). The internal consistency for these items was high (α = 0.89). Two additional items (labeled as OWS-2) used simpler wordings: “My weight is very important for me right now” and “My shape is very important for me right now.” Participants indicated their answers by sliding a bar on the computer screen from 0 (disagree) to 100 (agree). The internal consistency for these two items was high (α = 0.87). Although all four items were theoretically expected to measure the same construct, the reliability analysis of the four items showed questionable internal consistency (α = 0.64). Consequently, we decided to analyze the two sets of items separately. For both sets, responses on the items were averaged, with higher scores indicating higher overvaluation of weight and shape.

##### Motivational Salience of Body-Related Stimuli

A computerized version of a color-naming interference task was presented on OpenSesame software, version 3.2.7. Kafkaesque Koffka (Mathôt et al., 2012). The stimuli consisted of 10 fat-related words (thighs, stout, hips, fat, heavy, plump, bum, overweight, thick, chubby), 10 thin-related words (slim, lean, skinny, flat, light, small, petite, slender, thin, waist), 10 neutral words matched to the fat words (plank, window, closet, desk, sofa, poster, carpet, towel, pillow, lamp), and 10 neutral words matched to the thin words (curtain, bed, door, clock, table, vase, painting, cloth, chair, blanket). The neutral words were matched to the body-related words on number of characters and frequency of word use. The words were displayed in one of four colors: red, yellow, blue, green.

Participants were instructed to indicate the color in which each word was displayed by pressing the response key in the corresponding color. Participants were instructed to respond as quickly and accurately as possible. The word “Error” was presented after incorrect responses and remained on the screen until the correct response was made. Each trial was separated by a 300 ms interval. Before starting the actual task, participants conducted one practice block of 12 stimuli that were not used in the test trials. Following Ostafin and Feyel (2019) and recommendations of Cox et al. (2006), the main task was presented in blocked format. That is, one block of body-related stimuli was presented (i.e., thin or fat), which was followed by the corresponding block of neutral stimuli. Then, the other block of body-related stimuli was presented (i.e., thin or fat), which was again followed by the corresponding block of neutral stimuli. Participants were randomly assigned to one of the two block orders (experimental condition: thin first *n* = 32, fat first *n* = 33; control condition: thin first *n* = 30, fat first *n* = 33). As each word was presented twice in each of the four colors, every block consisted of 80 items.

#### Secondary Outcome Measures

##### Self-Reported Salience of Thin-Related and Fat-Related Goals

To assess the self-reported salience of thin-related and fat-related goals, two items were presented: “At this moment, how important is it for you to try to become thinner?” and “At this moment, how important is it for you to avoid becoming fat?” Participants indicated their answers by sliding a bar on the computer screen from 0 (not at all) to 100 (very). The two items were averaged, with higher scores indicating stronger goals related to weight and shape. The internal consistency for these items was α = 0.79.

##### Intention to Diet

Intention to diet was assessed with two items: “Right now, I plan to limit the amount of food that I eat” and “Right now, I plan to more often avoid the high calorie foods that I like.” The items were rated and scored in the same manner as the aforementioned goal items, with higher scores indicating stronger intention to diet. The internal consistency for these items was α = 0.78.

#### Meaning and Control Manipulations

Participants were randomly assigned to one of two conditions, both of which involved reading an essay, summarizing the essay (maximum of 25 words), and then receiving further essay-specific instructions (the exact essays and instructions can be found in the Supplementary Material). In the experimental condition, participants read an essay about the importance of living an authentic life vs. a life of conformity to the group (361 words). After summarizing the essay, participants were asked to list three values that are intrinsically important to them and one behavior they could exert over the next month that would be in accordance with each value. In the control condition, participants read an essay about computers. After summarizing the essay, participants were instructed to list three examples of how they use computers and what technological advances might occur in the next 5 years that will influence the way they use computers. The meaning manipulation was adapted from previous research, which found the manipulation to lead to a greater sense of purpose (Ostafin and Proulx, 2019).

#### Manipulation Check

Two items were used to assess state activation of valued life goals: “This essay reminds me that I have aims in my life that are worth striving for” and “This essay reminds me that I have life goals that compel me to keep going.” Participants rated the items from 1 (strongly disagree) to 5 (strongly agree). The responses were averaged, with higher scores indicating higher activation of valued life goals. The internal consistency was high (α = 0.92). Two items were used to assess the level of engagement: “This essay is interesting” and “I was engaged while reading this essay.” The items were rated and scored in the same manner as the aforementioned items, with higher scores indicating stronger engagement. The internal consistency for these items was α = 0.79.

#### ED Symptoms

For descriptive purposes the Eating Disorder Examination Questionnaire (EDE-Q; Fairburn and Beglin, 2008) was administered prior to the manipulation. The EDE-Q assesses ED symptoms over the last 28 days and provides a global measure of the severity of ED pathology. Participants indicate their answers on a scale between 0 (no days/not at all) and 6 (every day/markedly). Responses on the 22 items are averaged, with higher scores indicating higher ED psychopathology (cf. Aardoom et al., 2012). The internal consistency in the current sample was high (α = 0.91).

#### Life Meaning

The Multidimensional Existential Meaning Scale (MEMS; George and Park, 2017) was used to assess participants' life meaning prior to the manipulation. In line with the tripartite view, the MEMS assesses the three sub-constructs of life meaning, using 15 items that are rated on a scale ranging from 1 (very strongly disagree) to 7 (very strongly agree). The responses on the items are averaged, with higher scores indicating higher life meaning. The internal consistency was high (α = 0.93).

### Procedure

The study was approved by the Ethical Committee Psychology of the University of Groningen (17347). Data collection was conducted in small groups of one to three participants seated in private cubicles. Participants received instructions about the study protocol and provided written informed consent. To conceal the actual purpose of the study, participants were told that associations between happiness, mood, and concentration were studied. After participants completed baseline questionnaires (including the demographic variables, EDE-Q, and MEMS), practice trials of the color-naming interference task were completed. Participants were then randomly assigned to either the experimental (*n* = 65) or control condition (*n* = 63). Once the manipulation was completed, participants completed post-manipulation measures (including the manipulation check, overvaluation of weight and shape, secondary outcome measures, and eight bogus items in line with the cover story) and notified the experimenter, who then started the color-naming interference task. Following completion of the study, participants were thanked, debriefed, and dismissed.

### Data Reduction

We planned to calculate the color-naming interference effect on basis of reaction times and error rates. However, the error rate was too low to be able to compute a meaningful measure of interference (0.5% of the total data). Therefore, the color-naming interference effect was only indexed by reaction times. In line with Ostafin and Feyel (2019), responses faster than 200 ms or slower than 2,000 ms were considered to reflect anticipations or distractions and were omitted from all analyses (0.07% of data). After the errors were removed, two difference scores were computed. The thin-neutral difference score was calculated as the mean reaction time of the thin trials, minus the mean reaction time of the matched neutral trials. The fat-neutral difference score was calculated as the mean reaction time of the fat trials, minus the mean reaction time of the matched neutral trials. To index the reliability of the interference measures Spearman-Brown corrected split-half correlations were computed. For the thin-neutral difference score *r* = 0.69; for the fat-neutral difference score *r* = 0.65. For both indices, the estimate of reliability was satisfactory for this type of performance-based measure in the context of between condition comparisons (cf. Dresler et al., 2012).

### Statistical Analyses

To test whether participants in the meaning condition would show lower self-reported overvaluation of weight and shape than participants in the control condition, a MANOVA was performed with OWS-1 and OWS-2 as dependent variables, and Condition (experimental vs. control) as the independent variable. Power calculations show that the power of this analysis was 0.70 to detect medium-sized effects with α = 0.05, and 0.98 to detect large effects. To test whether, compared to controls, participants in the experimental condition would show less interference for body-related words, a MANOVA was conducted with the two reaction time difference scores (thin-neutral and fat-neutral) as dependent variables, and Condition and Order (thin first vs. fat first) as independent variables. Power analyses indicate that the power of this analysis was 0.68 to detect medium-sized effects and 0.98 to detect large effects. Regarding the secondary outcome measures, independent *t*-tests were performed for self-reported salience of weight and shape goals and intention to diet, with Condition as independent variable. Power calculations show that the power of these analyses was 0.88 to detect medium-sized effects and 0.99 to detect large effects.

## Results

### Descriptives

#### Participant Characteristics

For descriptive purposes participant characteristics are presented in Table 1.

**Table 1:** Group characteristics.

	**Experimental condition (*****n*** **=** **65)**		**Control condition (*****n*** **=** **63)**	
	***Mean***	***SD***	***Mean***	***SD***
Age	20.78	2.76	21.37	3.04
BMI	24.31	7.25	22.77	3.06
EDE-Q	3.03	1.08	2.86	1.00
MEMS	4.47	1.07	4.80	0.95

*BMI, Body Mass Index; EDE-Q, mean score on the Eating Disorder Examination Questionnaire (range 0–6); MEMS, mean score on the Multidimensional Existential Meaning Scale (range 1–7). Although BMI seems to be higher in the experimental condition, this apparent difference was driven by outliers*.

#### Manipulation Check

The state measures of activated life goals and level of engagement were used as manipulation checks. When checking the assumptions for the analyses, outliers were detected. Because excluding the outliers did not significantly change the results, analyses using all participants are reported. An independent *t*-test showed that the meaning manipulation more strongly activated valued life goals (*M* = 3.94, *SD* = 0.97) compared to the control condition (*M* = 2.30, *SD* = 0.89), with a large effect size [*t*_(126)_ = 9.93, *p* < 0.001, *d* = 1.76]. The meaning essay also elicited more engagement (*M* = 4.12, *SD* = 0.90) than the control essay (*M* = 3.72, *SD* = 0.86), with a moderate effect size [*t*_(126)_ = 2.58, *p* = 0.011, *d* = 0.45].

To explore the values that participants reported, we classified all values (*N* = 195) into different categories based on the classification system of Schwartz and Bilsky (1987). Participants most often stated values from the prosocial domain (*n* = 109, 55.9%), as “honesty,” “helping others,” “having relationships with others,” and “being empathic.” The self-direction domain followed (*n* = 51, 26.2%), with values as “creativity” and “personal growth.” Related to the achievement domain participants mentioned values as “doing well at university,” “dedication,” and “hard working” (*n* = 12, 6.2%); related to the enjoyment domain participants mentioned values as “being happy” and “pleasure” (*n* = 11, 5.6%). Values from the other domains were rarely mentioned (security *n* = 8, 4.1%; maturity *n* = 3, 1.5%; restrictive-conformity *n* = 1, 0.5%; social power *n* = 0, 0.0%). However, it is important to keep in mind that these results might have been biased to a certain degree, as frequently mentioned values as “honesty,” “creativity,” and “helping others” were given as example items in the instructions (see Supplementary Material).

### Effect of Manipulation on Self-Reported Overvaluation of Weight and Shape

The multivariate test for Condition was not significant [Wilks' Λ = 0.991, *F*_(2, 125)_ = 0.55, *p* = 0.576], indicating that there was no meaningful difference in current overvaluation of weight and shape between participants in the experimental (OWS-1 *M* = 3.29, *SD* = 1.81; OWS-2 *M* = 51.59, *SD* = 30.65) and control condition (OWS-1 *M* = 3.21, *SD* = 1.65; OWS-2 *M* = 54.32, *SD* = 28.10).

### Effect of Manipulation on Color-Naming Interference

Data from five participants were omitted from the analyses for the following reasons: four participants did not have data from the color-naming interference task (due to a computer error) and one participant fell asleep during the experiment. When calculating the interference scores, two outliers were found (mean +3 *SD*; for the thin-neutral difference score one participant from the control condition, for the fat-neutral difference score one participant from the experimental condition). Since the results did not change significantly when the two outliers were excluded from the analyses, analyses including the two outliers are reported. The multivariate test for Condition was not significant [Wilks' Λ = 0.998, *F*_(2, 118)_ = 0.134, *p* = 0.874], indicating no difference in interference (thin vs. neutral stimuli; fat vs. neutral stimuli) between the experimental and control condition, when controlling for order. Furthermore, the multivariate test for the interaction between Condition and Order was not significant [Wilks' Λ = 0.998, *F*_(2, 118)_ = 0.126, *p* = 0.876]. The multivariate test for Order, however, was significant [Wilks' Λ = 0.939, *F*_(2, 118)_ = 3.86, *p* = 0.024]. This indicates that interference by thin words was greater when participants started with the thin-block, and interference by fat words was greater when participants started with the fat-block. See Table 2 for the mean reaction times per condition for each block. See Supplementary Material for the interference scores per order and condition.

**Table 2:** Mean reaction time (in milliseconds) and interference score per condition.

	**Experimental condition (*****n*** **=** **62)**		**Control condition (*****n*** **=** **61)**	
	***Mean***	***SD***	***Mean***	***SD***
RT, neutral stimuli	680	77	701	82
RT, matched to thin stimuli	682	76	704	83
RT, matched to fat stimuli	677	82	698	86
RT, body-related stimuli	694	81	713	85
RT, thin stimuli	694	80	712	91
RT, fat stimuli	695	88	714	84
Interference thin	11	31	8	37
Interference fat	18	40	15	34

*RT, reaction time; Interference thin, reaction time difference score thin-neutral trials; Interference fat, reaction time difference score fat-neutral trials. Positive scores for Interference scores indicate greater interference by body-related stimuli*.

### Secondary Measures

When checking the assumptions, four outliers were detected and assumptions of normality were violated for the self-reported salience of thin-related and fat-related goals. When the outliers were excluded from the analysis, visual inspection confirmed that normality improved and results showed that the self-reported salience of thin-related and fat-related goals were lower for participants in the experimental condition (*M* = 61.19, *SD* = 29.74) than for participants in the control condition (*M* = 69.88, *SD* = 17.57), [*t*(105.48) = −2.00, *p* = 0.048, *d* = 0.36]. However, when all participants were included, participants in the experimental condition (*M* = 61.19, *SD* = 29.74) no longer significantly differed from participants in the control condition (*M* = 65.75, *SD* = 23.37), [*t*(120.88) = −0.97, *p* = 0.337, *d* = 0.17]. Finally, we found no significant difference with respect to intention to diet between participants in the experimental (*M* = 45.26, *SD* = 29.98) and control condition (*M* = 48.89, *SD* = 27.81), [*t*(126) = −0.71, *p* = 0.480, *d* = 0.13].

### *Post-hoc* Analyses

To examine the criterion validity of the interference effects as an implicit measure of current weight and shape concerns we *post-hoc* computed Pearson's correlations among the interference indices and relevant dependent variables (Table 3). Supporting the validity of the interference measure as an implicit index of current weight and shape concerns, color-naming interference by fat words was positively related to self-reported overvaluation of weight and shape (OWS-1 *r* = 0.19, *p* = 0.037; OWS-2 *r* = 0.20, *p* = 0.025), self-reported salience of goals related to weight and shape (*r* = 0.19, *p* = 0.035), and intention to diet (*r* = 0.21, *p* = 0.017). No significant correlations were found between interference by thin words and relevant dependent variables.

**Table 3:** Pearson's correlations among interference indices and other dependent variables.

**Variable**	***n***	**1**	**2**	**3**	**4**	**5**	**6**
1. Interference thin	123		−0.03	0.04	−0.02	0.03	−0.05
2. Interference fat	123			0.19^*^	0.20^*^	0.19^*^	0.21^*^
3. OWS-1	128				0.72^**^	0.62^**^	0.58^**^
4. OWS-2	128					0.81^**^	0.70^**^
5. Goals W and S	128						0.66^**^
6. Intention to diet	128						

*Interference thin, reaction time difference score thin-neutral trials; Interference fat, reaction time difference score fat-neutral trials; OWS, overvaluation of weight and shape; Goals W and S, self-reported salience of goals related to weight and shape*.

* *p < 0.05*;

** *p < 0.01*.

Finally, to explore whether the current manipulation was more impactful for individuals with predisposing characteristics, Pearson's correlations among study variables were *post-hoc* calculated for both conditions separately (Table 4). In both conditions, baseline MEMS was positively related to the degree in which the essays were effective in activating valued life goals (experimental condition *r* = 0.36, *p* = 0.003; control condition *r* = 0.35, *p* = 0.004). This might indicate that the manipulation check potentially captures trait life meaning, rather than state activation of life goals. Furthermore, specifically in the experimental condition, baseline MEMS was positively related to the degree in which the manipulation elicited engagement (*r* = 0.27, *p* = 0.028), indicating that the meaning-related essay elicited more engagement in participants who already possess a sense of life meaning. In the experimental condition, baseline MEMS was also negatively related to post-manipulation self-reported overvaluation of weight and shape (OWS-1 *r* = −0.28, *p* = 0.023). Furthermore, for both conditions it appeared that participants who reported more ED symptoms prior to the manipulation, also reported higher post-manipulation overvaluation of weight and shape (OWS-1 experimental condition *r* = 0.68, *p* < 0.001; control condition *r* = 0.42, *p* = 0.001; OWS-2 experimental condition *r* = 0.55, *p* = 0.003; control condition *r* = 0.40, *p* = 0.001), salience of goals related to weight and shape (experimental condition *r* = 0.50, *p* < 0.001; control condition *r* = 0.39, *p* = 0.002), and intention to diet (experimental condition *r* = 0.45, *p* < 0.001; control condition *r* = 0.34, *p* = 0.007). Finally, in the experimental condition, the degree in which the manipulation was effective in activating valued life goals correlated negatively with self-reported salience of goals related to weight and shape (*r* = −0.28, *p* = 0.024). This indicates that the salience of goals related to weight and shape reduced more for participants whose valued life goals were activated to a higher degree.

**Table 4:** Pearson's correlations among study variables, for both conditions separately.

**Condition**	**Variable**	***n***	**1**	**2**	**3**	**4**	**5**	**6**	**7**	**8**	**9**	**10**	**11**	**12**
Experimental	1. Age	65		0.03	−0.05	0.28^*^	−0.08	−0.20	0.06	−0.41^**^	0.04	0.07	−0.04	0.04
	2. BMI	65			0.13	−0.06	−0.04	−0.07	0.14	−0.01	0.18	0.21	0.19	0.05
	3. EDE-Q	65				−0.13	−0.02	0.02	0.25	0.20	0.68^**^	0.55^**^	0.50^**^	0.45^**^
	4. MEMS	65					0.36^**^	0.27^*^	−0.10	−0.05	−0.28^*^	−0.20	−0.23	−0.21
	5. MC Goals	65						0.66^**^	−0.01	0.18	−0.21	−0.18	−0.28^*^	−0.12
	6. MC Engagement	65							−0.06	0.13	−0.14	−0.13	−0.19	−0.06
	7. Interference thin	62								−0.04	0.18	−0.03	−0.05	0.04
	8. Interference fat	62									0.19	0.27^*^	0.23	0.28^*^
	9. OWS-1	65										0.73^**^	0.64^**^	0.62^**^
	10. OWS-2	65											0.83^**^	0.74^**^
	11. Goals W and S	65												0.68^**^
	12. Intention to diet	65												
Control	1. Age	63		0.19	−0.15	0.21	0.20	0.29^*^	0.15	−0.03	−0.13	−0.05	−0.05	−0.01
	2. BMI	63			0.10	−0.00	−0.08	−0.00	0.02	−0.04	−0.23	−0.23	−0.11	−0.29^*^
	3. EDE-Q	63				0.04	−0.04	0.00	−0.01	−0.12	0.42^**^	0.40^**^	0.39^**^	0.34^**^
	4. MEMS	63					0.35^**^	0.15	0.17	−0.05	−0.01	0.12	0.07	−0.06
	5. MC Goals	63						0.43^**^	0.14	−0.05	0.06	0.07	−0.03	−0.01
	6. MC Engagement	63							0.14	−0.26^*^	−0.19	−0.14	−0.10	−0.13
	7. Interference thin	61								−0.02	−0.08	−0.01	0.12	−0.12
	8. Interference fat	61									0.18	0.11	0.15	0.13
	9. OWS-1	63										0.72^**^	0.60^**^	0.53^**^
	10. OWS-2	63											0.77^**^	0.66^**^
	11. Goals W and S	63												0.62^**^
	12. Intention to diet	63												

*BMI, Body Mass Index; EDE-Q, mean score on the Eating Disorder Examination Questionnaire; MEMS, mean score on the Multidimensional Existential Meaning Scale; MC Goals, Manipulation check state activation valued life goals; MC Engagement, Manipulation check level of engagement; Interference thin, reaction time difference score thin-neutral trials; Interference fat, reaction time difference score fat-neutral trials; OWS, overvaluation of weight and shape; Goals W and S, self-reported salience of goals related to weight and shape*.

* *p < 0.05*;

** *p < 0.01*.

## Discussion

The aim of the present study was to examine the influence of life meaning on ED symptoms and motivational processes in women at risk for developing EDs. We expected that a brief meaning manipulation involving reflection on and commitment to valued life goals would lead to lower self-reported current overvaluation of weight and shape and less color-naming interference of body-related words. Our findings did not corroborate these hypotheses, as we found no differences between participants in the meaning and control condition in self-reported weight and shape concerns or color-naming interference of body-related words. Thus, the present study did not provide evidence for a causal influence of life meaning on the current overvaluation of weight and shape in a high-risk group.

Previous research has shown a correlation between life meaning and ED symptoms in clinical samples (Fox and Leung, 2009; Marco et al., 2017, 2019). The current findings failed to support the hypothesis that life meaning would have a causal influence on ED variables. The current findings are also not in line with previous findings that a similar meaning manipulation lowered the color-naming interference of alcohol-related stimuli in a sample of undergraduate students who reported regular alcohol consumption (Ostafin and Feyel, 2019). It should be acknowledged, however, that this earlier effect was only found with regard to the interference effect indexed by error rates, whereas in the current study error rates were too low to be meaningfully used to index interference effects.

When interpreting the current findings, four factors need to be considered that might have contributed to the failure to find support for the causal influence of life meaning on current overvaluation of weight and shape in a high-risk group. A first explanation for the current null findings might relate to using overvaluation of weight and shape, also known as the ‘core psychopathology' of EDs (Fairburn et al., 2003), as an outcome measure. According to Veale (2002), over-valued ideas are so important to the individual that they become integrated into their identity and are held very strongly. In line with this, theory and empirical findings suggests that in individuals with EDs the construct of overvaluation of weight and shape is stable, hard to change in treatment, and closely tied to self-esteem (Garner and Bemis, 1982; Cooper and Fairburn, 1993; Killen et al., 1994; Fairburn et al., 2003). Furthermore, the overvaluation of weight and shape and the extent to which it generalizes to all domains of self-esteem is known to differentiate individuals with EDs from restrained and unrestrained eaters (McFarlane et al., 2001; Wilksch and Wade, 2004). Thus, because weight and shape concerns appear so strongly ingrained in the identity of individuals with EDs, it is likely that the exposure to the meaning manipulation needs to be stronger to even temporarily affect their overvaluation of weight and shape. Second, it is therefore also possible that our meaning manipulation was not effective in activating intrinsically valued life goals strongly enough, to lower participants' overvaluation of weight and shape in the experimental condition. Although the effect of the manipulation on state activation of goals in the present study (*d* = 1.76) was at least as large as the effect of the same manipulation in a prior study (Ostafin and Proulx, 2019), the manipulation is rather short and only seems to address the purpose component of life meaning, while neglecting the components of comprehension and significance (George and Park, 2016; Martela and Steger, 2016). Therefore, future research should test whether intensifying the present manipulation (e.g., repeating the current task over several days or weeks) or creating a more overarching intervention that addresses all three components of life meaning increases the effectiveness of the manipulation and, in the end, successfully lowers overvaluation of weight and shape. Furthermore, as the instructions of the current manipulation were fairly directive in nature (see the Supplementary Material), it is possible that the manipulation was more effective in individuals who were intrinsically motivated to reflect on their life goals. Similarly, as previous research showed that the level of pretreatment motivation is predictive of treatment outcome in individuals with eating disorders (Clausen et al., 2013), the current manipulation might also be more effective in participants who are intrinsically motivated to change. Thus, for future research it is important to examine whether these factors potentially moderate the intervention effects. Finally, it is important to note that for the two main analyses regarding overvaluation of weight and shape, our sample size secured a power of, respectively, 0.70 and 0.68 (with α = 0.05) for finding medium sized effects. Therefore, it cannot be ruled out that we might have missed relatively weak effects of life meaning on overvaluation of weight and shape.

The current findings appear to be inconsistent with those of Ostafin and Feyel (2019). However, two important (methodological) differences between both studies should be taken into account when interpreting the results. First, the color-naming interference task in the study of Ostafin and Feyel (2019) consisted of 180 trials, while participants in the present study had to complete a total of 320 trials. The large number of trials in the present study might have hampered the detection of manipulation effects, as previous studies showed that participants habituated to the content of target words (Green and Rogers, 1993) and interference of body-related words decreased across the course of color-naming interference tasks (Green et al., 1994). This idea seems to be supported by the significant multivariate test for Order (see Supplementary Material), indicating that interference by thin words was greater when participants started with the thin-block, and interference by fat words was greater when participants started with the fat-block. However, it is important to note that the positive associations between the fat-neutral interference score and self-reported overvaluation of weight and shape, self-reported salience of thin-related and fat-related goals, and intention to diet (see Table 3), not only corroborate the relevance of the interference index as a measure of individual differences, but also supports its criterion validity and relevance for ED symptoms. Second, the error rate in the present study (0.5% of all data) was much lower than in the study of Ostafin and Feyel (2019) (5.3% of all data). Perhaps this discrepancy is related to differences in personality traits in individuals with EDs (e.g., pathological perfectionism and over-control) vs. individuals with addiction (e.g., impulsivity and risk-taking) (O'Hara et al., 2015). The latter idea seems to be supported by the reaction time patterns of both studies, which showed that participants in the current study were generally slower to respond (i.e., RT neutral stimuli *M* = 690 ms, RT body-related stimuli *M* = 703 ms) than participants in the study of Ostafin and Feyel (2019) (i.e., RT music stimuli *M* = 635 ms, RT alcohol stimuli *M* = 629 ms), which could indicate that participants in the current study responded less impulsive possibly leading to longer reaction times and fewer errors.

With respect to the secondary outcome measures, the findings of the present study were mixed. Regarding the self-reported salience of thin-related and fat-related goals, results showed that participants in the experimental condition reported lower salience of thin-related and fat-related goals after the manipulation, compared to participants in the control condition. However, since the effect disappeared when the outliers were retained in the analysis, this finding should be interpreted with caution. Furthermore, participants in the experimental condition did not significantly differ in their intention to diet from participants in the control condition. Regarding the *post-hoc* analyses, we found that the more participants already possess a sense of meaning in life, the easier it is to elicit engagement in a meaning-related essay. Participants with higher baseline meaning in life also reported lower post-manipulation overvaluation of weight and shape and were, in line with previous research (Steger et al., 2009), generally older. Additionally, an older age was related to less interference by fat words in the experimental condition. Finally, in line with previous research on addictive behaviors showing that increased accessibility and commitment to non-alcohol life goals reduced the motivational pull of alcohol-related cues (Palfai and Weafer, 2006; Ostafin and Feyel, 2019), *post-hoc* analyses showed that the more valued life goals were activated by the meaning manipulation, the lower the salience of goals related to weight and shape.

Despite the strengths of the present study (using an experimental design to investigate the relationship between life meaning and ED symptoms, including a manipulation check, and using a performance-based implicit measure of weight and shape concerns), at least four limitations should be taken into consideration when interpreting the results. First, as elaborated on earlier, our findings possibly indicate that overvaluation of weight and shape is a stable construct which is hard to change using a short meaning manipulation. Therefore, future studies should include validated state measures of ED-related motivation. Another possibility is to include other ED-related outcome variables to test whether more short-term ED-related behaviors can be activated in the absence of more automatic cognitive change. Second, during the meaning manipulation participants had to report three values that reflect their true selves and one behavior they could exert over the next month that would be in accordance with each value. Although it is possible that some types of values were associated with stronger effects, the current study was not designed to test these kinds of hypotheses. Thus, it might be interesting for future research to qualitatively examine the listed values and accompanying behaviors, and to quantitatively examine whether certain values are associated with stronger effects. As a third limitation it should be mentioned that, although participants were randomly assigned to the experimental or control condition, the type of reimbursement between conditions was not equally distributed. Therefore, it cannot be ruled out that this overrepresentation of participants receiving course credits in the experimental condition might have affected the current findings. Since the present study was not designed to test such differences and lacked sufficient power to do so reliably, future studies should examine whether type of reimbursement affects the impact of the current meaning manipulation. Finally, in the current study we relied on a non-clinical (analog) sample. It can therefore not be ruled out that meaningful differences would have occurred when studying a clinical sample.

Despite several studies showing associations between life meaning and ED symptoms, we did not find any positive effects of a meaning manipulation on current overvaluation of weight and shape, either in terms of self-report indices or a performance-based measure (i.e., color-naming interference effect of thin and fat-related stimuli). Therefore, our study did not provide evidence for a causal role of life meaning in current weight and shape concerns in a high-risk group. Ultimately, one of the goals of the present study was to gain an initial understanding of potential treatment options focusing on life meaning in individuals with EDs. There are already a few extensive psychotherapies that focus on creating a life worth living based on personal values, such as Acceptance and Commitment Therapy (ACT; Hayes et al., 2006), which have been successfully applied in the treatment of ED pathology (Manlick et al., 2013). However, our findings suggest that we first need to examine the relationship between life meaning and ED symptoms in more detail, before we can consider implementing brief meaning manipulations in clinical practice.

## Data Availability Statement

The raw data supporting the conclusions of this article will be made available by the authors, without undue reservation.

## Ethics Statement

The current study involving human participants was reviewed and approved by the Ethical Committee Psychology of the University of Groningen. The participants provided their written informed consent to participate in this study.

## Author Contributions

SD: formal analysis, writing—original draft, writing—review, and editing. KG: conceptualization, methodology, supervision, writing—review, and editing. BO: conceptualization, writing—review, and editing. PJ: writing—review & editing. All authors contributed to the article and approved the submitted version.

## Conflict of Interest

The authors declare that the research was conducted in the absence of any commercial or financial relationships that could be construed as a potential conflict of interest.
