# Supplementary material for: The Causal Influence of Life Meaning on Weight and Shape Concerns in Women at Risk for Developing an Eating Disorder
Source: Front Psychol. 2021 Feb 11;12:593393. doi: 10.3389/fpsyg.2021.593393 (PMC7905029; doi:10.3389/fpsyg.2021.593393)
Supplement: Supplementary file 1 [file Data_Sheet_1.pdf]

Supplementary Material

10.3389/fpsyg.2021.593393

1 Supplementary Data

Instructions experimental condition

Please take your time to read the following essay. Your comprehension of the essay will be assessed after you are finished reading.

<u>Essay</u>
This essay about life's meaning is written by a British university professor:

My inquiry into what makes life worth living began in my youth. It was a time of deep searching and questioning. The question of life's meaning has been central to my life and has led me to spend some thirty years researching, thinking and writing about this topic. If I were to summarize what I have learned into simple and plain words, I would say something along the following lines:
There is something special about human beings. The complexity of our brains means that we can transcend our time and place in ways that no other animals can. And we can know ourselves. Our capacity for self-awareness means that we can know what is unique and special about our lives as individuals.

An important consequence of self-knowledge is that it creates a choice, one that can sometimes be difficult, between:

1) Living a life that consists of following the trends and opinions of the group. By this, I mean doing so in an automatic, conforming way (i.e., without a sense of really thinking about whether the group's values are in line with your authentic values).
2) Living a life that is authentic to ourselves. By this, I do not mean acting on every impulse or simply being defiant towards others. What I mean is following your deepest values…knowing yourself and being true to the deepest parts of who you are. Although there are exceptions, these values are often not understood or appreciated by others.

Being authentic isn't easy because there is a great deal of subtle and overt pressure to follow the group. In addition, we are all motivated to fit in and don't like to "rock the boat" – to make others uncomfortable by acting in ways that contradict the group's values. But what I'd most like to tell you is that your time is limited, so don't waste it living someone else's life. Don't get trapped by what other people want. Don't let the noise of others' opinions drown out your own inner voice. And, most important, have the courage to follow your heart and intuition. They somehow already know what you truly want to become. Everything else is secondary.

<u>Instruction</u>
With just one sentence, please write the main point of the essay you read (maximum of 25 words):

As the essay mentioned, it is often difficult to disregard the values of the group because of pressure to conform and because of our own concerns about pleasing others and fitting in. Additionally,

following our own, inner voice – basing our behavior on our own unique sense of what is important – can make other people uncomfortable.

For this part we will ask you to write about three values that are intrinsically important to you. When we say ‘values’, we are thinking of something that is different from ‘goals’. Goals are something specific that can be accomplished – e.g., making a beautiful painting. In contrast, values are more general and are something you strive after but can’t completely achieve – e.g., being a creative person. For each of the three values, please complete the following two steps.

1. In your own words, list three values that reflect your true self – your own, inner voice. These can be anything, for instance:

- Creativity
- Honesty
- Personal growth
- Helping others
- Relationships with family or friends

The main thing is that these are values that you hold deeply, irrespective of what others think is important.

2. For each of the three values, write about one thing you could do over the next month to behave in accordance with it. For example, if your value is helping others, you could write that you’re going to help an elderly person with grocery shopping.

Again, this can be anything – what is important is that this behavior is about acting in accordance with what is most important to you rather than about pleasing others. Try to describe as specific as possible what you can do in the next month, that is in line with your value.

Instructions control condition

Please take your time to read the following essay. Your comprehension of the essay will be assessed after you are finished reading.

Essay

This essay about computers is written by a British university professor:

Computers are able to recognize, remember, store, and manipulate many forms of abstract symbols, including every human language and the mathematical languages of the sciences. In fact, the words you are looking at right now were put through a machine which stored them electronically and which allowed me, the author, to manipulate them several times before they were definitely stored on the computer.

But even though all these words went through an 'electronic memory' which kept every key-stroke for as long as I wanted, the computer never understood a word of this text. A computer does not comprehend what is stored in its 'memory' any more than a book in the library understands what it contains. Nevertheless computers can become quite clever with language. Human language can be broken down into its component parts - phrases, words, and individual letters of the written form. A

computer can be taught to 'translate' a word into another human language by being told that they are equivalent.

But this is all being done mathematically with ones and zeros in the processor of the electronic brain. In contrast to humans, the computer does not understand what it is doing or the symbols it manipulates. The computer cannot grasp the leaps of thought we call metaphors (for instance, what sense would it make of a "leap" of thought?). Computers are limited because they are not persons. They have no human experience as a background for understanding what was said. Rather they must depend entirely on the mechanical structures put into their 'brains' by human beings.

Instruction

With just one sentence, please write the main point of the essay you read (maximum of 25 words):

As the essay mentioned, computers have become quite "smart" in the past years. In the western world, most people use them on a daily basis at work as well as at home.

For this part we will ask you to write about three ways how you use computers (in your daily life).

For each of the three ways, please complete the following two steps.
In your own words, list three ways that you use computers personally. It does not matter how they help society in general or other people around you, just how you use it.
For each of the three ways, write what technological advances you think will occur in the next 5 years.

2 Supplementary Figures and Tables

Supplementary Table 1

Interference score (in milliseconds) as a function order

	Thin first (n = 59)		Fat first (n = 64)	
	Mean	*SD*	*Mean*	*SD*
Interference thin	18	36	1	30
Interference fat	16	44	17	29

Note. Interference thin = reaction time difference score thin-neutral trials, Interference fat = reaction time difference score fat-neutral trials. Positive scores for Interference scores indicate greater interference by body-related stimuli.

Supplementary Table 2

Interference score (in milliseconds) as a function of order and condition

	Experimental Condition		Control Condition	
	Thin first (n = 31)	Fat first (n = 31)	Thin first (n = 28)	Fat first (n = 33)

	Mean	*SD*	*Mean*	*SD*	*Mean*	*SD*	*Mean*	*SD*
Interference thin	18	34	4	27	19	39	0	33
Interference fat	18	51	18	26	14	37	17	32

Note. Interference thin = reaction time difference score thin-neutral trials, Interference fat = reaction time difference score fat-neutral trials. Positive scores for Interference scores indicate greater interference by body-related stimuli.
